# Epigenetic aging of semen is associated with inflammation

**DOI:** 10.1080/15592294.2024.2436304

**Published:** 2024-12-05

**Authors:** Junxi Feng, Liudmilla Rubbi, Reza Kianian, Jesse Nelson Mills, Vadim Osadchiy, John Tucker Sigalos, Sriram Venkata Eleswarapu, Matteo Pellegrini

**Affiliations:** a Molecular, Cell and Developmental Biology, University of California Los Angeles, Los Angeles, CA ,USA; b Division of Andrology, Department of Urology, David Geffen School of Medicine at UCLA, Los Angeles, CA, USA

**Keywords:** Epigenetics, male infertility, aging, semen, sperm

## Abstract

Male infertility has been a primary cause of global infertility, affecting 8–12% of couples worldwide. Previous studies have shown that semen quality decreases with advanced aging with an increased presence of inflammatory cells. In this study, we examined changes in the epigenome across a diverse cohort that includes both fertile and infertile men. We also compare the age-associated changes in semen to those observed in buccal swabs in order to characterize differences in epigenetic aging across diverse tissues. We found that variations in the semen methylome associated with aging are linked to inflammatory genes. Many age-associated sites are demethylated with advanced aging and are associated with the activation of inflammatory pathways. By contrast, we do not observe age-associated changes in inflammatory genes in buccal swab methylomes, which instead are characterized by changes to bivalent promoters. Our findings highlight the potential of epigenetic markers as indicators of male reproductive health.

## Background

The analysis of semen is of interest for its relevance to male infertility and reduced fertility with age. Semen analysis, serum sex hormone evaluation, karyotyping, Y chromosome microdeletion assays, sperm DNA fragmentation testing, empiric medical therapy, surgical intervention, and assisted reproductive technology have become mainstays in the diagnosis and management of male factor infertility and age-associated reduced fertility. Despite improvements in the evaluation and treatment of infertility over the last few decades, the vexing challenge of unexplained male factor infertility persists in cases where routine semen analysis yields normal results [1]. Therefore, a more precise understanding of the etiologies of male factor infertility and a better understanding of age-related changes to semen is needed.

Male infertility is often marked by a decline in sperm quality, a concern that escalates with advanced aging [2]. In older males, germ cells become increasingly vulnerable to genetic mutations, a concept termed advanced paternal aging, conferring increased risk for psychiatric and developmental disorders, such as autism spectrum disorder (ASD), congenital heart disease, and attention deficit disorder [3–5]. The decline in sperm quality may be partly attributed to the presence of inflammatory cells in the semen [6–8]. As a result, there has been significant interest in studying the effect of molecular factors that impact sperm.

To better dissect human spermatogenesis and gain insights into the mechanisms of male infertility and age-associated changes in semen, novel multi-omic technologies at molecular resolution have been employed, including whole-genome bisulfite sequencing, single-cell RNA-seq, whole-exome sequencing, and transposase-accessible chromatin using sequencing [9]. Among these omic approaches, DNA methylation is one of the most extensively explored epigenetic modifications, although a deeper understanding of the regulation of DNA methylation in the male germline may augment the clinical evaluation of male infertility. This epigenetic modification is characterized by the addition of methyl groups to the C5 position of cytosine and occurs preferentially at cytosine-phosphate-guanine (CpG) dinucleotides. These CpG sites are typically concentrated in regions known as CpG islands and are found near gene promoter regions [10]. The methylation status of CpG islands plays a crucial role in modulating gene regulation: when the CpG sites within a gene promoter are unmethylated (hypomethylated), the DNA is more accessible to transcription factors that initiates transcription and thus promotes gene expression. In addition to promoter regions, methylation status of enhancers can also affect gene expression. Specifically, active enhancers are partially demethylated, facilitating the protein binding that enhances the transcription of associated genes [11]. Additionally, different epigenetic ‘clock’ models have been developed to study the association between DNA methylation profiles and chronological age in most somatic tissues [12,13]. While epigenetic clock estimators showed remarkable predictive accuracy for determining the epigenetic age of somatic tissues, their performance dropped significantly when applied to predict the epigenetic age of sperm [12]. The poor predictive power can be rationalized by the fact that the epigenome of germ cells is fundamentally different from the epigenome of somatic cells [14].

More recently, several epigenetic clocks for sperm have been developed to study the association between sperm epigenetic age and clinical factors such as semen parameters and pregnancy outcomes, as well as the discovery of differentially methylated sites (DMSs) [15–18]. Although studies have been conducted to elucidate age-related epigenetic alterations of human sperm cells and further develop age prediction models, comparatively less emphasis has been placed on investigating age-associated changes in semen. Several cell types compose semen, including leukocytes, germ cells, and epithelial cells [19]. The diversity of cell-type compositions in semen highlights the importance of accounting for their relative abundance when assessing age-associated changes [20,21].

In this study, we collected semen and buccal swabs from a cohort of 83 men. Conventional semen analysis and baseline health characteristics were obtained. The DNA methylation profiles were measured using targeted bisulfite sequencing of DNA samples collected from both sources. We first performed cell-type deconvolution analysis to measure the cell-type composition in semen and buccal swabs. To study the effect of multiple factors that could affect the epigenome, we built multifactor models to model the effect of age and different cell-type compositions on the DNA methylation profiles. Our findings indicate that inflammatory processes are associated with the aging of semen. Moreover, our findings suggest that DNA methylation may eventually serve as a biomarker for evaluating male reproductive health.

## Materials and methods

### Overview

**Figure 1.**
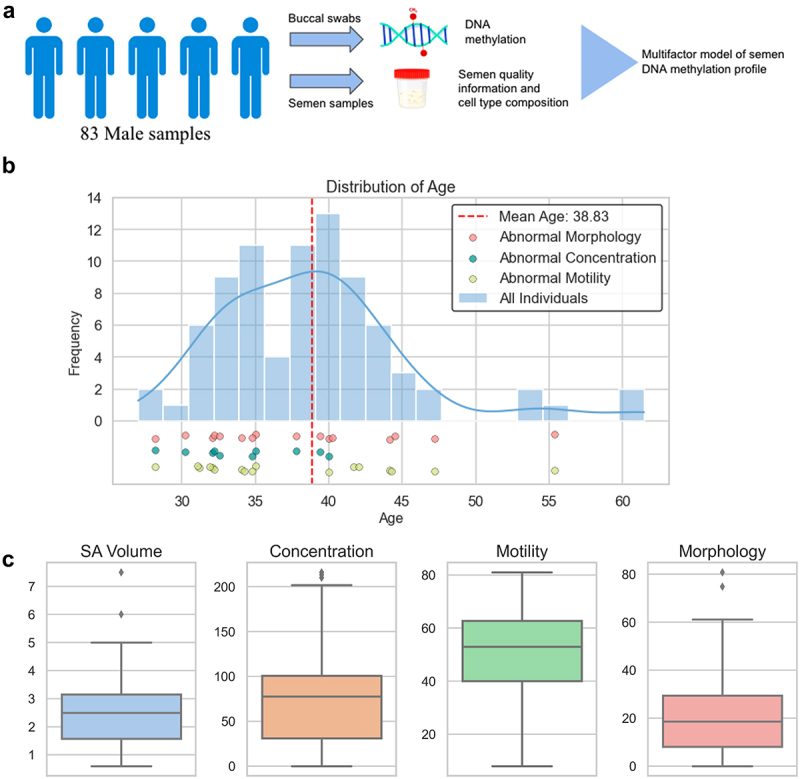
Schematic overview and data distribution of the samples.

We collected the semen and buccal swabs from 83 male patients and performed targeted bisulfite-sequencing to obtain their DNA methylation profiles. We then estimated the cell-type composition of the samples and constructed a multifactor model, incorporating DNA methylation data from both tissue types to explore how methylation patterns relate to patient age and cell composition (Figure 1a). Finally, we conducted Cistrome and GTEx analysis by filtering for statistically significant methylation sites from the model (see Results).

(a) Schematic overview of the study. (b) Age distribution of the collected samples and the dots below indicate individuals with abnormal sperm quality. Red dots represent individuals with abnormal sperm morphology, blue dots represent individuals with abnormal sperm concentration, and yellow ones represent individuals with abnormal sperm motility. (c) Bar plot that shows the distribution of semen characteristics: semen volume (mL), sperm concentration (M/mL), sperm motility (%), and sperm morphology (% by Kruger strict criteria).

### Study population and biospecimen collection

With institutional review board approval (IRB #21–000714), we prospectively recruited males aged ≥18 years who presented to an academic reproductive urology clinic from July 2022 through June 2023. Recruited participants sought consultation either for an initial fertility evaluation or for vasectomy consultation after biological paternity. All participants provided written informed consent for collection of clinical data and biospecimens. Participants were excluded if there was a recent history of acute illness. Semen specimens were collected following 2–7 days of abstinence. Contemporaneous buccal swabs were obtained from each participant. All specimens were obtained prior to any procedural or pharmacologic intervention for fertility. For DNA methylation analysis, a swab was taken from each well-mixed semen specimen. Buccal and semen swabs were stored at −80°C until processing for DNA methylation analysis.

### Semen analysis

Conventional quantitative semen analysis was performed according to World Health Organization 5^th^ Edition criteria using a calibrated SQA-Vision Automated Semen Analyzer (Medical Electronic Systems, Encino, CA). Samples demonstrating oligozoospermia or azoospermia were independently evaluated using high-powered microscopy. Semen volume, concentration, total motility, strict morphology (Kruger), and pH are reported.

### Targeted bisulfite sequencing (TBS-seq)

Buccal swabs and semen samples were incubated overnight at 50°C before DNA extraction. DNA was extracted using reagents from DNA Genotek (Stittsville, Ontario, Canada). Targeted bisulfite sequencing (TBS-seq) was applied to characterize the methylomes of 83 samples. The details of the protocol have been previously described [22]. In summary, 500 ng of extracted DNA were used for TBS-seq library preparation. The DNA was fragmented, followed by end repair, dA-tailing, and adapter ligation using the NEBNext Ultra II Library Prep Kit with custom pre-methylated adapters from IDT. Pools of 16 purified libraries were hybridized to the biotinylated probes according to the manufacturer’s protocol. Captured DNA was treated with bisulfite prior to PCR amplification using KAPA HiFi Uracil+ (Roche) with the following conditions: 2 min at 98°C; 14 cycles of 98°C for 20 sec, 60°C for 30 sec, 72°C for 30 sec; 72°C for 5 min; hold at 4°C. Library QC was performed using the High-Sensitivity D1000 Assay on a 2200 Agilent TapeStation. Pools of 96 libraries were sequenced on a NovaSeq6000 (S1 lane) as paired-end 150 bases.

The probes used in the capture can be found in Supplementary File 1. They contain regions identified as having significant associations with multiple traits, including age in EWAS studies.

## Methylation matrix assembly

We aligned the bisulfite sequencing data using BSBolt (v1.5.0) [23]. First, the GRCh38 human reference genome was indexed with the BSBolt Index command and the FASTQ files were aligned to the reference genome using the BSBolt Align command. Post-alignment processing including PCR duplicate removal was performed using SAMtools (v1.15) [24]. Next, the BSBolt CallMethylation command was used to generate CGmap files before the methylation matrix aggregation. We only kept methylation sites above a minimum read depth coverage of 40, which resulted in a methylation matrix with 72,000 CpG sites and 83 samples. Finally, we imputed the matrix with the BSBolt Impute command to fill in the missing values using the k-Nearest Neighbors (kNN) method. The methylation matrices for both the semen and buccal swab samples were constructed in a similar manner.

## Cell-type deconvolution

Human semen is composed of a variety of cell types, including sperm, leukocytes, and epithelial cells [25]. Variations in cell-type abundance have a significant impact on DNA methylation levels. Therefore, it is critical to account for cell-type effects when performing DNA methylation analysis [20]. We used CELLFi, a reference-based tool that applies a non-negative least-squares regression model, to estimate the fraction of each reference cell type (https://github.com/dmontoya09/CEllFi_v01). We used whole-genome bisulfite sequencing (WGBS) data of healthy human tissues from [26] for the cell-type reference methylome. In total, we used WGBS data from blood T lymphocytes (including naive T cells [*n* = 3] and CD8+/CD4+ effector/central memory T cells [*n* = 11]), blood granulocytes (*n* = 3), and prostate epithelium (*n* = 4). In addition, we used WGBS data of sperm (*n* = 3) from [27]. We also filtered the entire reference methylome data based on the sites from our targeted bisulfite sequencing probes (72000 CpG sites). The fraction of each cell type in each semen sample was estimated by CELLFi using the default parameters.

The same cell-type deconvolution technique was also applied to buccal samples. The cell-type reference methylome also came from [26]. We used WGBS data from blood T lymphocytes (including naive T cells [*n* = 3], and CD8+/CD4+ effector/central memory T cells [*n* = 11]), blood B lymphocytes (including B cells [*n* = 3] and memory B cells [*n* = 2]), blood granulocytes (*n* = 3), blood monocytes (*n* = 3), blood NK cells (*n* = 3), and prostate epithelium (*n* = 4). We observe that the WGBS data of prostate epithelium is similar to that of buccal epithelium, so for convenience we continued to use WGBS data of prostate epithelium as a reference.

## Multivariate multiple regression model

To account for the effect of multiple factors on DNA methylation, we built a multivariate multiple regression model, which we refer to as the multifactor model. For an individual sample, we modeled its seminal DNA methylation level at a particular CpG site as a linear combination of the associated factors weighted by coefficients. We can formulate the relationship as follows:

$$M^{\mathrm{obs}}=X\cdot \beta$$

Here, $M^{\mathrm{obs}}$ represents the observed methylation matrix of individuals by CpG sites, Xrepresents the multifactor phenotype matrix of individuals by phenotypic factors, and β represents the coefficient matrix of phenotypic factors by CpG sites. Similar to a linear regression, the objective is to find the least squares solution with a set of coefficients that minimize the squared error. In this case, we applied the Moore-Penrose pseudoinverse technique to find the solution. First, we estimated the coefficient matrix as $=X^{†}\cdot M^{\mathrm{obs}}$, where $X^{†}$ is the Moore-Penrose pseudoinverse of X. Once the coefficient matrix was obtained, we computed the pseudoinverse again to predict the multifactor matrix as $X^{\mathrm{pred}}=M^{\mathrm{obs}}\cdot \beta^{†}$, and predict the methylation matrix as $M^{\mathrm{pred}}=X\cdot \beta$.

We preprocessed the factor matrix by removing correlated factors to avoid multicollinearity. The T-cell composition and granulocyte composition were highly correlated to the sperm abundance, and thus removed. The final model included three factors: sperm cell abundance, prostate epithelial cell composition, and age. All factors were scaled to a range of 0 to 1. A constant term was added to the multifactor matrix. In addition, a batch effect term was added to account for technical variability, since the samples were collected in two batches.

To avoid overfitting, we applied LOOCV by excluding one test sample in each iteration and training the model on the remaining samples. Within each iteration, we first estimated the coefficient matrix β using the formula described above (see Multivariate multiple regression model), and then predicted the multifactor matrix $X^{\mathrm{pred}}.$Finally, we measured the Pearson correlation between $X^{\mathrm{pred}}$ and $X^{\mathrm{obs}}$ to evaluate the model performance.

A nearly identical procedure was applied to the DNA methylation analysis of buccal specimens, except that the final multifactor model included only a constant term, age, epithelial cell composition, and batch effect term.

## Methylation site selection

We defined three statistical ‘filters’ to select the significant CpG sites associated with each factor from the model. The first filter is **High Correlation**: we calculated the correlation between the predicted methylation matrix $M^{\mathrm{pred}}$and the observed methylation matrix $M^{\mathrm{obs}}$ (see Multivariate multiple regression model), and only kept the sites with an absolute value of the correlation greater than or equal to 0.5. The second filter is **Statistical Significance**: we constructed a multiple linear regression model similar to the multifactor model, where the dependent variable was the methylation status, and the independent variables were the factors and a constant term. The *p*-values from the multiple linear regression model were estimated and then adjusted for multiple hypothesis testing using the Benjamini Hochberg procedure. We kept the sites with *p*-values less than or equal to 0.05. The last filter is **the Highest Coefficient**: we restricted the methylation sites associated with a specific factor to be the ones with the highest absolute value of the coefficient among all factors in β.

After applying the filtering processes mentioned above, we chose the top 200 methylation sites based on the adjusted *p*-values for a particular factor. We examined the sign of the coefficient matrix β and further separated the sites into those with positive coefficients and those with negative coefficients. A negative correlation indicates the loss of DNA methylation, whereas a positive correlation means the gain of methylation with increasing values of the factor. We performed the filtering procedure on the seminal and buccal swab multifactor models separately.

## Cistrome and GTEx analysis

After selecting the significant sites associated with each factor, we analyzed the genomic coordinates of the significant sites using the Cistrome Data Browser (Cistrome DB) [28]. Similar samples from Cistrome DB were used to identify overlaps with the peak sets we defined, and transcription factors with a significant binding overlap were returned. We also performed a functional enrichment analysis using Cistrome-GO [29]. The significant GO terms are summarized in the Results section. The top 50 genes ranked by adjusted RP score (i.e., proximity to promoters) from Cistrome were further analyzed using tissue-specific expression data from GTEx [30] to identify the gene expression level across different tissues.

## Results

### Characterization of the semen methylome

Our study aims to explore the relationship between DNA methylation of semen and factors that drive changes in methylation including semen cell-type composition and chronological age. We collected semen and buccal swab samples from 83 male patients. The semen was characterized based on sperm concentration, motility, and morphology. Around 88% of the samples came from men between ages 30 and 45 years old, 2% of the samples are less than 30 years old, and 10% of the samples are greater than 45 years old; individuals with abnormal semen parameters were evenly distributed across the sample age range, and the leukocyte count was measured as binary (Figure 1b). The body mass index and semen parameters for the study cohort are provided in Figure 1c and Table 1. The descriptive statistics of the infertile and vasectomy samples are also shown in Supplementary Figure S1A and Figure 1b.

**Table 1.** Age, body mass index, and semen analysis parameters for the study cohort.

	Mean ± S.D.	Range
Age (y)	38.8 ± 6.4	27.0–61.5
Body mass index (BMI)	26.6 ± 6.0	18.7–61.6
Semen analysis		
Semen volume (mL)	2.5 ± 1.3	0.1–7.5
Sperm concentration (M/mL)	63.9 ± 57.0	0–216.1
Total sperm motility (%)	46.3 ± 21.3	0–81
Strict morphology (%)	20.1 ± 16.7	0–80.8
Semen pH	8.2 ± 0.24	8.0–8.8

We measured the DNA methylation of each sample using targeted bisulfite sequencing. This approach has certain advantages over the widely used DNA methylation arrays, reduced representation bisulfite sequencing (RRBS) or whole-genome bisulfite sequencing (WGBS), as it allows for the selection of regions of interest in the genome and the generation of high coverage data to obtain accurate estimates of DNA methylation at those sites. By contrast, arrays have fixed probes, and RRBS of WGBS tend to generate lower coverage datasets. The limitation of our approach is that the number of targets we select is limited compared to other methods, but our selection criteria allows us to enrich biologically interesting sites based on the mining of prior datasets. The probes we used in our targeted bisulfite sequencing assay were chosen from different sources: some were selected from the EWAS Atlas [31] and represent regions with significant EWAS hits across multiple studies; others were collected from epigenetic clocks, such as the Horvath [12], Hannum [13], GrimAge [32] and PhenoAge [33] clocks; and another set contained sites with cell-type-specific DNA methylation regions (see Supplementary File 1 with list of probes). Our targeted panel leads to the generation of a methylation matrix of 72,000 CpG sites across 83 individuals, with a minimum coverage of 40 and average coverage of 74.

### Cell-type analysis of DNA methylation data

**Figure 2.**
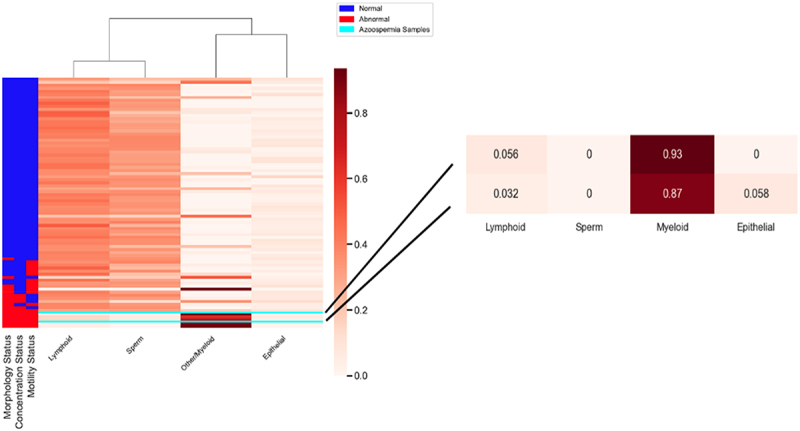
Cell-type composition of semen.

It is widely recognized that cell-type heterogeneity can have a significant impact on DNA methylation levels. To account for cell-type heterogeneity in semen, we considered four major cell types, including sperm cells, prostate epithelial cells, lymphoid cells, and an unknown fraction of cell types that we suspect to have characteristics of myeloid cells. For the buccal swab specimens, we included both epithelial and immune cells. We obtained the whole-genome bisulfite profiles of these cell types from [26] and [27]. We conducted cell-type deconvolution for the semen samples using a non-negative least-squares regression approach based on CpG methylation calls (Figure 2). Not surprisingly, the deconvolution results suggest an inverse relationship between sperm cell abundance and the other cell types. Given that the cell-type compositions are estimated from DNA and that sperm cells are haploid while the other types of cells are diploid, our estimates do not directly measure the fraction of cells. Nonetheless, we identified two azoospermia samples that had zero sperm percentages and displayed a significant proportion of an unknown cell type with myeloid characteristics. These individuals have an impairment of sperm production with unidentified etiology.

Heatmap of cell-type composition for semen samples. The cell-type composition is computed using CELLFi. Semen analysis parameters of interest were sperm concentration, motility, and morphology. Two azoospermia samples displayed a significant proportion of an unknown cell type with myeloid characteristics.

## Identification of factors that influence DNA methylation in semen

**Figure 3.**
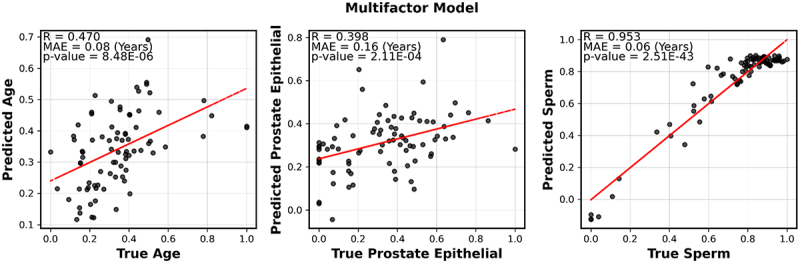
Multifactor model built on seminal DNA profile.

To jointly characterize multiple factors that influence DNA methylation in semen we used multivariate multiple regression. Semen DNA methylation was used to train a multifactor model incorporating a constant term, a batch effect term, age, sperm cell composition, and prostate epithelial cell composition. The lymphoid cell composition and other cell-type composition were highly correlated among themselves and with the sperm cell composition, hence these were removed to include only the two aforementioned cell types (Supplementary Figure S2A). We solve the model using the pseudoinverse method. Specifically, we first estimated the coefficient matrix as $=X^{†}\cdot M^{\mathrm{obs}}$, where $X^{†}$ is the Moore-Penrose pseudoinverse of the multifactor phenotype matrix X. Once the coefficient matrix was obtained, we computed the pseudoinverse again to predict the multifactor phenotype matrix as $X^{\mathrm{pred}}=M^{\mathrm{obs}}\cdot \beta^{†}$, and predict the methylation matrix as $M^{\mathrm{pred}}=X\cdot \beta$. We correlated the predicted values of age, prostate epithelial cell composition, and sperm cell composition with their actual values and found that all three factors have statistically significant correlations between their predicted and actual values (*p* < 0.05 and *R* ≥ 0.4) (Figure 3). Not surprisingly, the sperm cell composition of the samples inferred from deconvolution is well predicted by our model with a correlation coefficient of 0.953. The age prediction shows a moderate correlation coefficient of 0.47. Predictions on all three factors by our model show statistically significant results with very low *p*-values, indicating that the correlations observed are highly unlikely to be due to chance.

Correlation between the predicted value of factors (age, prostate epithelial cell composition of semen, sperm abundance of semen) against the true value of factors. The predictions were calculated using the multifactor model. MAE stands for the mean absolute error.

## Analysis of factor-associated methylation sites

In order to identify the DNA methylation sites that are most associated with the factors in our model, three filters were defined. First, we filtered for sites whose methylation level was highly correlated with the predicted methylation level $M^{\mathrm{pred}}$ generated from the multifactor model. Next, we selected methylation sites from the multivariate multiple linear regression model that had statistically significant coefficients. Third, we retained only methylation sites that had the highest absolute correlation coefficient among all factors from the coefficient matrix β. Finally, if there were more than 200 sites that met these criteria we chose the top 200 sites with the lowest adjusted *p*-values. To further analyze the factor-specific CpG sites, we divided the sites into positive and negative groups based on the sign of the coefficient in the multifactor model. Further details of the site selection procedure are described in the Methods section.

When we analyzed the selected sites associated with age in the seminal multifactor model, we found 83 methylation sites that had negative coefficients, which indicates the loss of methylation at these sites during aging. For sperm cell composition, we identified 48 sites with positive coefficients and the remaining 152 sites with negative coefficients. We analyzed the two groups of sites separately. For simplicity in naming, we will refer to the sites with negative coefficients for sperm cell composition as ‘negative sperm sites,’ and vice versa for the positive sites. We acknowledge that mature sperm cells are transcriptionally silent and do not actively transcribe genes during their final stages, yet we hypothesize that the epigenetic landscape, including DNA methylation and histone modifications, is established earlier in sperm development. This early setting of the epigenome may serve as a regulatory mechanism that persists into maturity, influencing sperm function and contributing to developmental processes after fertilization. As such, we believe that the methylation patterns we observe in sperm cells reflect these earlier epigenetic events, which may have significant biological relevance even after transcription has ceased.

We used Cistrome to identify transcription factors associated with the significant sites. Cistrome identifies enriched transcription factor (TF) binding sites within a set of input regions, allowing identification of potential regulators of methylation sites. The genomic coordinates of the factor-associated sites were input as peak sets to the Cistrome Data Browser (Cistrome DB). ChIP seq datasets from Cistrome DB were overlapped with the peak sets we defined, and transcription factors with a significant binding overlap were returned. Functional enrichment analysis was also performed using Cistrome-GO, and the significant Gene Ontology (GO) terms from cellular components, molecular functions, and biological processes were identified.

**Figure 4.**
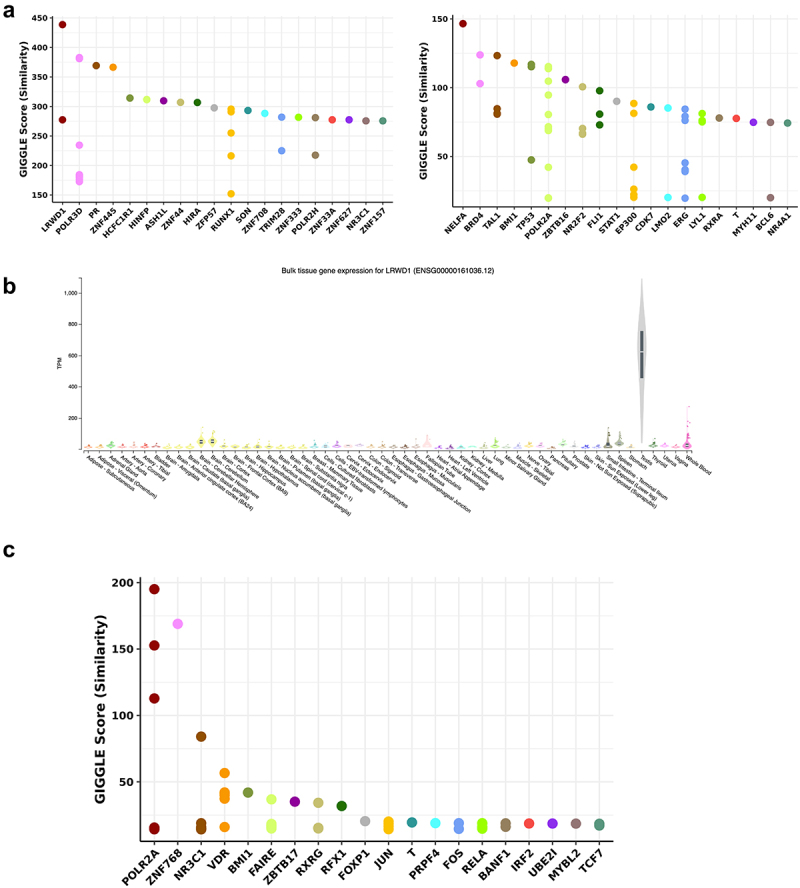
Transcription factors of methylation sites associated with sperm and age from the seminal multifactor model (generated from Cistrome).

For the positive sperm sites, the top three transcription factors were NELFA, BRD4, and TAL1 (Figure 4a). NELFA is part of the NELF protein complex and negatively regulates RNA polymerase II transcription elongation [34]. BRD4 is a kinase that phosphorylates RNA polymerase II and regulates transcription [35], and TAL1 is involved in multiple cellular processes including myeloid cell differentiation and positive regulation of cellular component organization [36]. The significant GO terms from molecular functions include cytokine activity, cytokine receptor binding, and chemokine activity. The significant GO terms from biological processes include immune response, defense response, and cytokine-mediated signaling pathway (Supplementary Table S1–2).

(a) Methylation sites associated with sperm from the multifactor model are divided into negative and positive groups based on their correlation with sperm cell composition. Each group of methylation sites is analyzed on Cistrome. Top ranking transcription factors associated with negative and positive sperm are shown in the plots. (b) LRWD1 expression level in TPM across human tissues. (c) Top ranking transcription factors associated with age are shown in the plot.

Among the negative sperm sites, the top three associated factors were LRWD1, POLR3D, and PR (Figure 4a). LRWD1 plays a major role in organization of heterochromatin structure in the somatic cells and is widely observed in human testis [37], which is also shown in Figure 4b. POLR3D is involved in RNA polymerase III transcription processes, and is associated with cell growth and proliferation [38]. The progesterone receptor (PR) gene is reported to cause male infertility [39]. The significant GO terms from cellular components include chylomicron and very-low-density lipoprotein particles. The significant GO terms from molecular functions include adenosine deaminase activity, receptor serine/threonine kinase binding, and deaminase activity (Supplementary Table S3–5).

For the negative age sites, the top three associated factors are associarted with POLR2A, ZNF768, and NR3C1 (Figure 4c). POLR2A is another RNA polymerase that is responsible for the transcription of a large fraction of protein-coding genes [40]. ZNF768 is a transcription factor [41] and the NR3C1 gene encodes glucocorticoid receptors and is found to regulate testicular functions [42]. The significant GO terms from cellular components include inflammasome complex, chylomicron, and cytoplasmic region. The significant GO terms from molecular functions include tau protein binding. The significant GO terms from biological processes include negative regulation of viral entry into the host cell, regulation of viral entry into the host cell, and cytokine-mediated signaling pathway (Supplementary Table S6–8).

**Figure 5.**
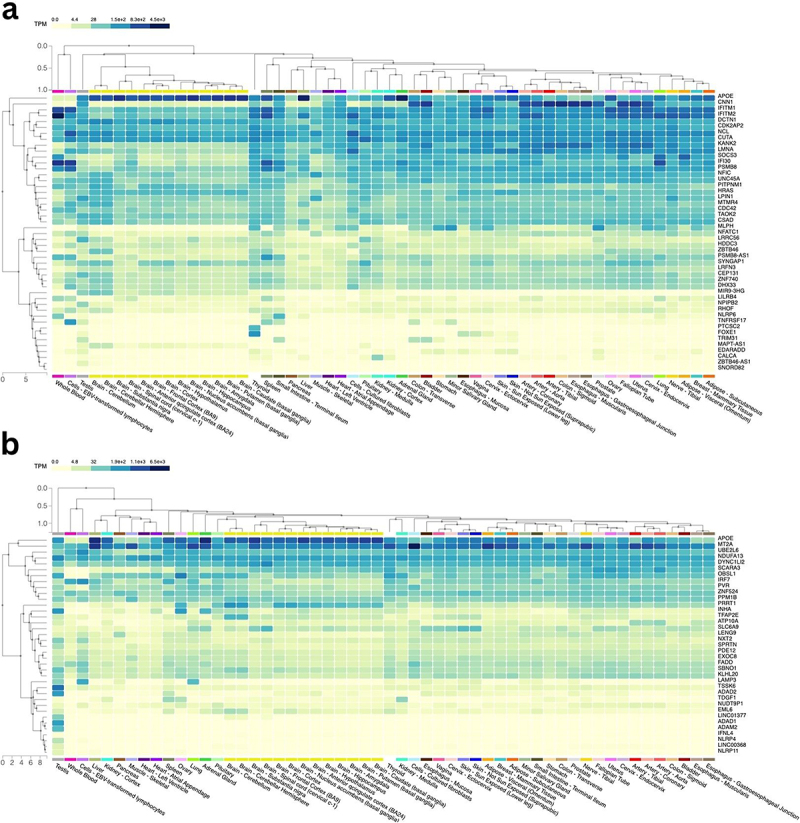
Gene expression in different tissue types (generated from GTEx).

To investigate the functional relevance of genes located near significant methylation sites, we examined their tissue-level expression using GTEx, a public database that enables querying gene expression levels across various human tissues. We chose the top 50 genes associated with negative age sites, positive sperm sites, and negative sperm sites. The genes are ranked by adjusted RP score calculated on Cistrome-GO, which measures the proximity to promoters. We then identified their gene expression level across different tissues. Among the genes associated with negative age sites, we observed expression across a broad range of human tissues. In particular, the genes that encode for interferon-induced transmembrane protein 1 (IFITM1), IFITM2, and IFITM3 were found to be specifically expressed in whole blood and EBV transformed lymphocytes (Figure 5a). In terms of the negative sperm sites, we noticed the high expression level of genes including testis-specific serine kinase 6 (TSSK6) and adenosine deaminase domain containing 1 (ADAD1), which were most highly expressed in human testis (Figure 5b).

(a) Gene expression associated with negative age methylation sites in the seminal multifactor model. (b) Gene expression associated with negative sperm methylation sites in the seminal multifactor model.

## Identification of factors that influence DNA methylation in buccal swabs

**Figure 6.**
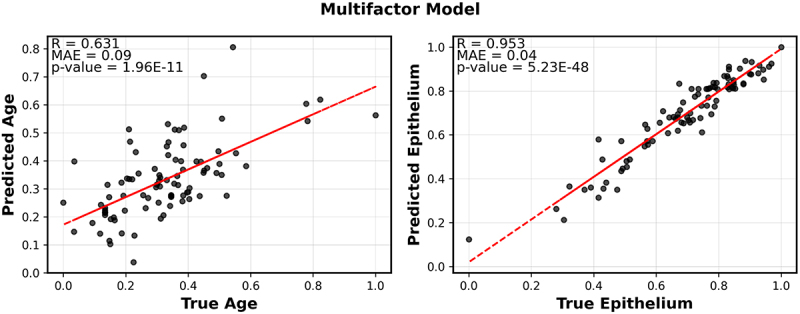
Multi-factor model built on buccal DNA profile.

DNA methylation in human buccal swabs has been shown to be a strong predictor of chronological age [43]. In this study, we sought to compare age-associated changes in buccal swabs and semen. We identified potential overlaps and differences between the significantly age-associated methylation sites in buccal swabs and semen to characterize common and divergent mechanisms of aging. To accomplish this goal, we performed a similar analysis for the buccal DNA methylation as for the semen methylation, which were collected from the same set of individuals. We developed a multifactor model for the buccal methylation sites. The buccal multifactor model was constructed using a similar approach to the semen multifactor model, excluding factors with high correlations (Supplementary Figure S2B). The buccal multifactor model incorporates a constant term, a batch effect term, and two phenotypic factors: age and epithelial cell composition. Both phenotypic factors exhibited statistical significance (*p* < 0.05) and strong correlation coefficients (*R* ≥ 0.6) when regressing the predicted values against the actual values (Figure 6).

Predicted vs. actual values of each variable in the multi-factor model for age, and epithelial cell composition.

We applied the same filtering procedure used in the semen multifactor model to select significant buccal methylation sites (see Method). We specifically analyzed the selected sites associated with age in the buccal multifactor model. We found 190 methylation sites associated with age with positive coefficients and 10 sites with negative coefficients. We analyzed the two groups of sites separately. For simplicity in naming, we will refer to the sites in the negative group associated with age as ‘negative age sites,’ and so on.

**Figure 7.**
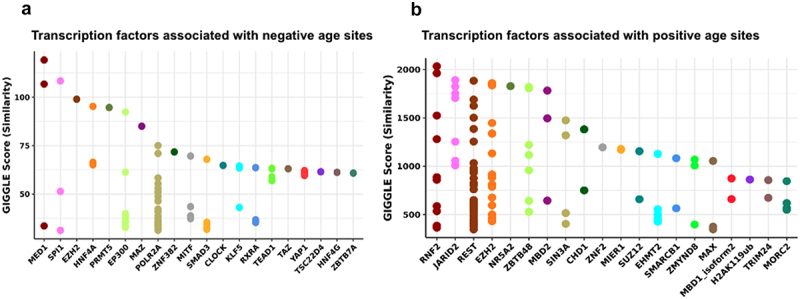
Transcription factors of methylation sites associated with sites from the buccal swab multi-factor model (generated from Cistrome).

We used Cistrome to conduct the analysis of the significant sites. For the negative age sites, the top three transcription factors were MED1, SPI1, and EZH2 (Figure 7a). Among other functions the mediator complex subunit 1 (MED1) is also involved in promoting oral mucosal wound healing and acts as a master regulator of epithelial cell fate [44,45]. The SPI1 gene encodes transcription factors that activate gene expression during myeloid and B-lymphoid cell development [46], and EZH2 is crucial for the maintenance of epithelial cell barrier integrity and an active participant that shapes the aging epigenome [47,48]. As for positive age sites, the top three transcription factors are associated with RNF2, JARID2, and REST (Figure 7b). The RNF2 and JARID2 genes are involved in transcriptional repression of genes involved in development and cell proliferation [49,50], and REST is a gene silencing transcription factor that is widely expressed during embryogenesis, and represses neural genes in non-neural tissues [51].

(a) Methylation sites associated with age from the buccal swab multi-factor model are divided into negative and positive groups based on their Pearson correlation with age. Each group of methylation sites is analyzed on Cistrome. Top ranking transcription factors associated with negative age are shown in the plots. (b) Top ranking transcription factors associated with positive age are shown in the plot.

## Histone modification analysis

**Figure 8.**
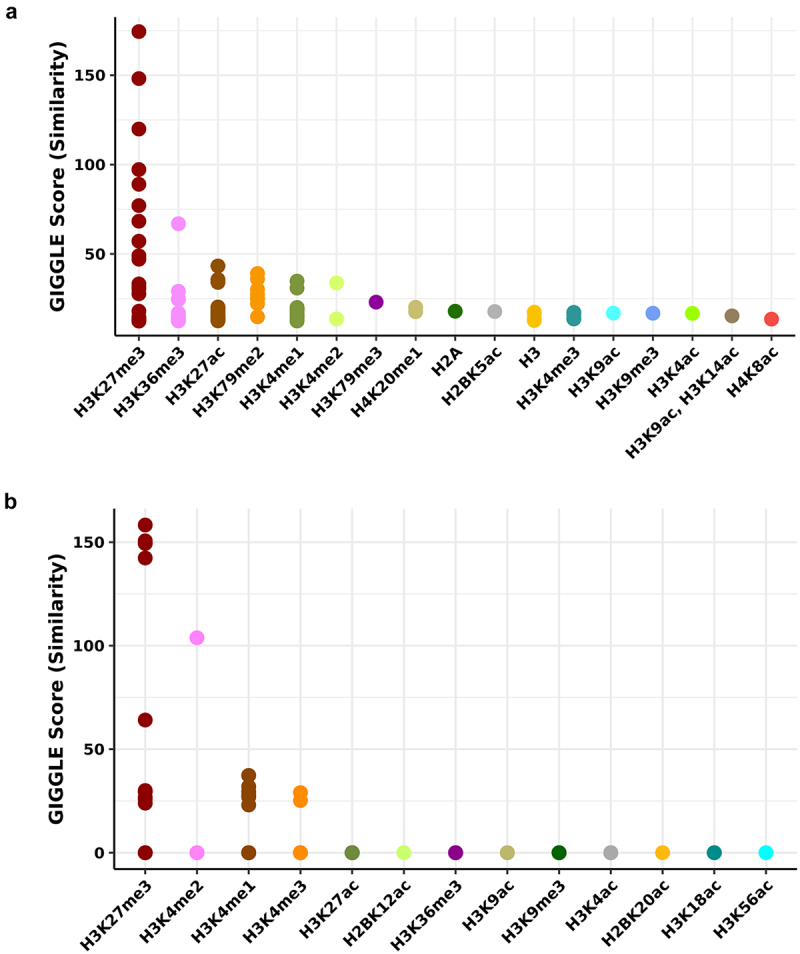
Histone modification associated with sites in semen and buccal swabs.

We also examined histone modification patterns of negative age sites from semen and buccal swabs using Cistrome DB to identify key differences in epigenetic regulation between semen and buccal swabs. H3K27me3 is ranked as the top histone modification for both semen and buccal swabs. Previous studies have shown that H3K27me3 is involved in silencing gene expression during embryonic stem cell differentiation [52,53]. We observe a distinct pattern of modification between the semen and buccal swabs specifically on H3K36me3 (Figure 8a,b). H3K36me3 is associated with regions that are transcribed, such as the gene bodies [54,55]. By contrast, the buccal swab sites contain H3K4me3, suggesting they are promoter sites. Since they contain both H3K27me3 and H3K4me3, they are likely enriched for bivalent promoters that repress genes in stem cells that are activated in a tissue-specific manner.

(a) Top ranking histone modifications are generated from Cistrome based on methylation sites that are associated negatively with age in seminal multi-factor models. (b) Similarly, top ranking histone modifications are generated from Cistrome based on methylation sites that are associated negatively with age in the buccal swab multi-factor model.

## Discussion

Our aims were to explore the influence of factors that impact DNA methylation in semen and to measure age-associated changes in the seminal methylomes. To this end, we first performed cell-type deconvolution analysis to determine the cell-type composition of semen. We estimated the abundance of spermatozoa, myeloid cells, lymphoid cells, and epithelial cells. Not surprisingly, we found that the presence of greater sperm DNA in semen is associated with lower immune cell composition. Several of the samples we profiled had been determined to have azoospermia, and we observed that these were indeed predicted to have very low sperm abundance, confirming our deconvolution methodology. The main components of semen in the azoospermia samples appear to have myeloid characteristics, which might be caused by the infiltration of neutrophils or monocytes in the semen.

Next, we asked if there were other factors than cell composition that were associated with DNA methylation levels. To answer this question, we developed a multifactor model that accounted for age, cell composition along with batch and constant terms. Finally, we asked which CpG sites were associated with each factor.

We found that the CpG sites that had a significant negative coefficient for sperm concentrations were strongly associated with the transcription factor LRWD1, a widely expressed protein in human testes. Due to the inverse relationship between DNA methylation and gene expression at promoters, the fact that the coefficient is negative suggests that these sites are associated with higher expression of proximal genes as sperm concentration increases. It is known that the expression level of LRWD1 is itself also regulated by the methylation status of the LRWD1 promoter, and is related to the modulation of spermatogenesis, sperm motility, and vitality [37]. Our results suggest that semen samples with higher sperm concentration are demethylated at LRWD1 binding sites compared to samples with low sperm concentration. In support of this notion, we find that about one-third of the genes that are closest to CpG sites with significant negative coefficients for sperm concentration are specifically expressed in testis. Some of the most highly expressed genes in human testis include TSSK6 and ADAD1, based on data from GTEx. In particular, testis-specific serine/threonine kinase (TSSK6) is known to regulate cell proliferation, and testis-specific adenosine deaminases (ADAD1 and ADAD2) play critical roles in germ cell differentiation [56,57].

Finally, we asked whether the genes with significant negative coefficients for sperm concentration were enriched for specific functions. We found that the enriched molecular functions include adenosine deaminase activity, and receptor serine/threonine kinase binding. Deaminase activity has been reported to affect the erasure of DNA methylation in mammalian primordial germ cells [58]. These results may indicate that the negative sperm sites identified by our multifactor model are associated with the maturation of sperm cells and potentially embryonic development.

We next investigated the age-associated changes in the DNA methylation of semen. We focused on the sites that had significant coefficients for age in our multifactor models, and found that these coefficients were predominantly negative, suggesting that the associated genes likely increase their expression with age. We found that the transcription factor binding that is enriched at these sites is NR3C1. The NR3C1 gene encodes the glucocorticoid receptor that is expressed in peritubular smooth muscle-like cells in adult testis, and is involved in sperm transport. Elevated glucocorticoid levels are linked to conditions such as Cushing syndrome and hormonal disruption, which can ultimately impair testicular functions and suppress male fertility [42,59–61]. Another enriched transcription factor is the vitamin D receptor (VDR). VDR is present in sperm and plays an important role in the maturation of human spermatozoa [62,63]. Finally, we found that genes closest to sites with significant negative coefficients for age included IFITM1, IFITM2, and IFITM3, which are interferon response genes that are activated following viral infections, such as the hepatitis C virus [64]. This result suggests that aging causes the loss of methylation in certain CpG sites that are associated with inflammatory responses, indicating an age-associated increase in the inflammatory state of semen.

To capture the DNA methylation pattern difference between germ cells and somatic cells along with aging, we performed a similar analysis on buccal swabs. We found that CpG sites with significant negative coefficients for age were associated with the transcription factor MED1, which plays a role in facilitating oral wound healing and regulates epithelial cell fate. Other transcription factors including SPI1 and EZH2 are also related to the regulation of epithelial cells and epigenomic aging. We found no overlap between the transcription factors identified in semen and buccal swabs. Similarly, there was no overlap between significant CpG sites associated with age in these two sample types (**Supplementary Figure S3**). Overall, our DNA methylation-based analysis suggests that aging biomarkers differ between semen and buccal swabs, reflecting a broader distinction between germ cells and somatic cells.

We recognize that the small sample size of only 83 samples is a limitation in this study. However, by analyzing the seminal DNA methylation of a cohort with different semen parameters can still provide valuable clinical insights into semen DNA methylation and epigenetic aging. Second, the reference profiles used in the cell-type deconvolution process may introduce bias, potentially overlooking certain cell types present in semen. Nonetheless, the use of well-established reference profiles available ensures that the most relevant and well-characterized cell types are accounted for, providing a reasonable approximation of the cell composition in semen.

In conclusion, we constructed a comprehensive model of DNA methylation in semen and buccal swabs that accounts for cell-type composition along with phenotypic factors. The computational method allows us to distinguish the methylation pattern of somatic cells and germline cells, providing key insights into sperm cell maturation and activity. By examining the significant CpG sites, we found a link between aging and increased inflammation in semen. As men age, demethylation at specific sites is associated with an increase in inflammatory gene expression, possibly causing sperm dysfunction and male infertility. In light of our findings, we believe that DNA methylation may offer new insights into the complex mechanisms of male infertility. Furthermore, we anticipate that such insights can be applied in clinical settings, potentially serving as reliable indicators of sperm fertility parameters and predictors of male infertility. This, in turn, could advance diagnostic procedures, providing clinicians with more accurate tools to assess and address male reproductive challenges.

## Ethics approval and consent to participate

Participant recruitment was approved by our institutional review board (IRB #21–000714). All participants provided written informed consent for collection of clinical data and biospecimens.

## Consent for publication

The content of this manuscript has not been previously published and is not under consideration for publication elsewhere.

## Availability of data and materials

The code for the analysis is available at https://github.com/JunxiFeng/Semen_DNAmeth_Analysis. The data described in this paper are accessible through GEO Series accession number GSE250598 (https://www.ncbi.nlm.nih.gov/geo/query/acc.cgi?acc=GSE250598).

## Supplementary Material

Supplementary File 1.xlsx

Supplementary_material.docx
