# Supplementary material for: Epigenetic aging of semen is associated with inflammation
Source: Epigenetics. 2024 Dec 5;19(1):2436304. doi: 10.1080/15592294.2024.2436304 (PMC11622584; doi:10.1080/15592294.2024.2436304)
Supplement: Supplementary_material.docx [file KEPI_A_2436304_SM2442.docx]

Supplementary Figure 1

**A**

**
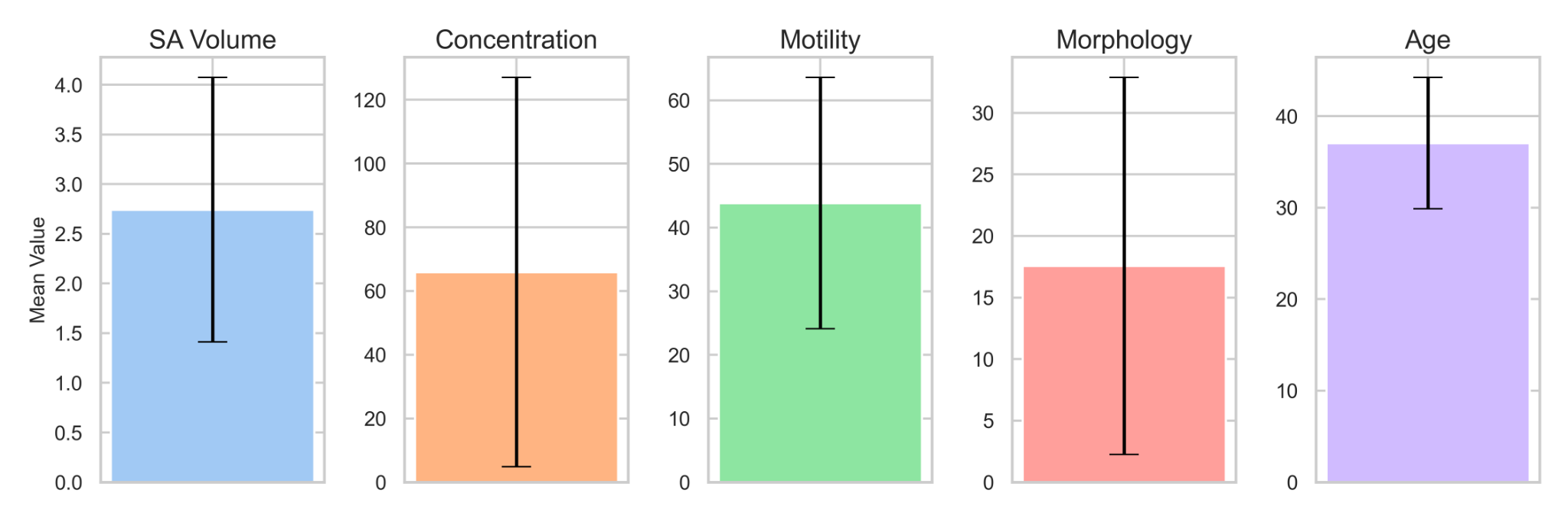
**

**B**

**
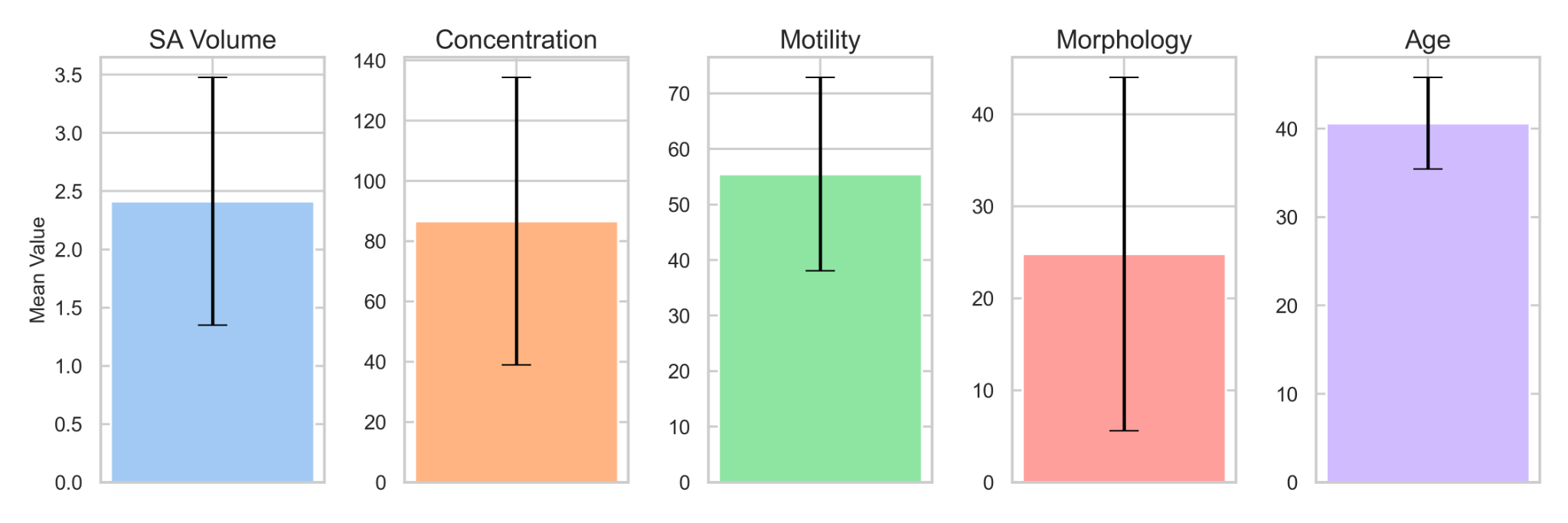
**

Supplementary Figure 2


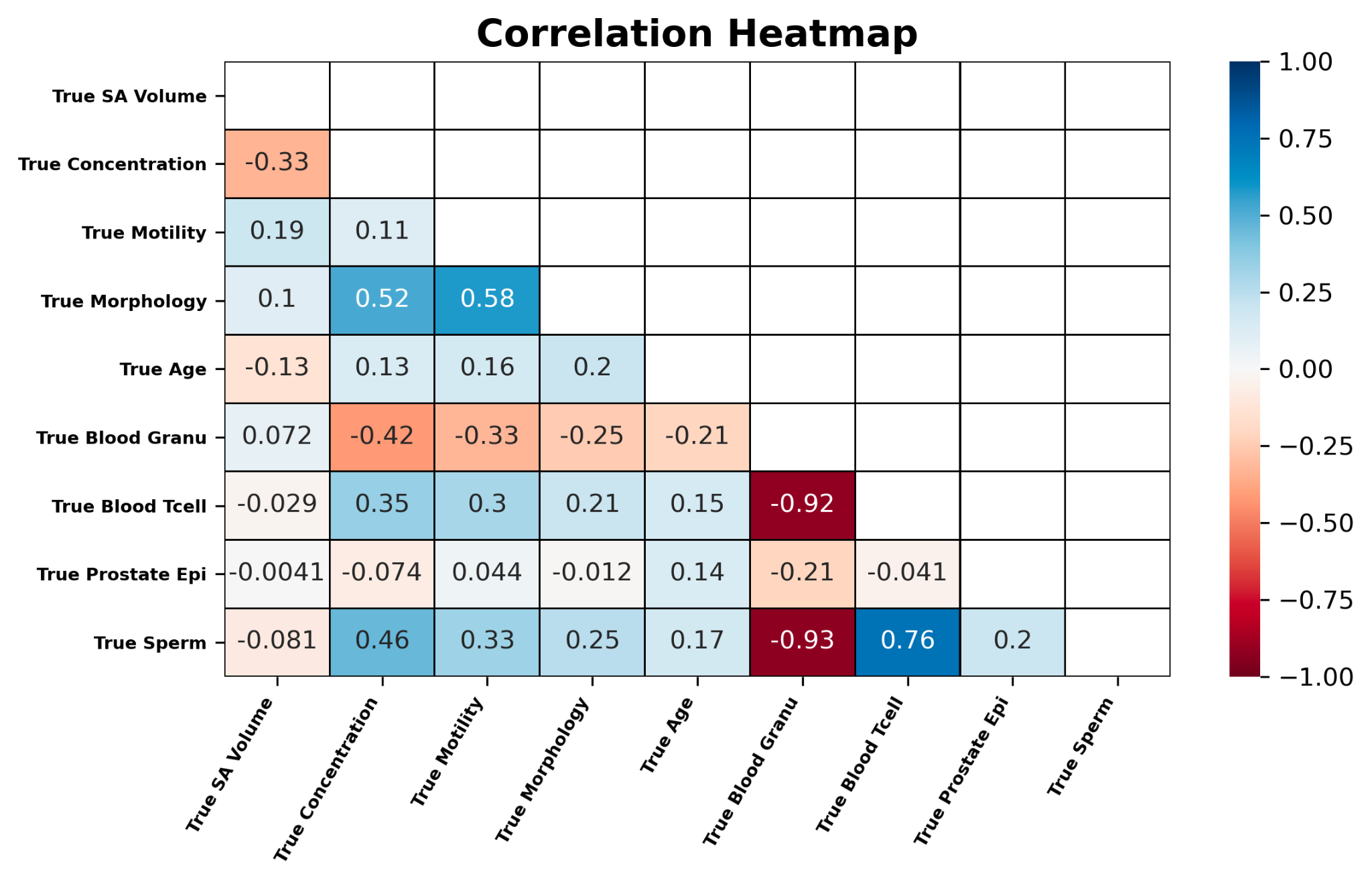


**A**

**B**
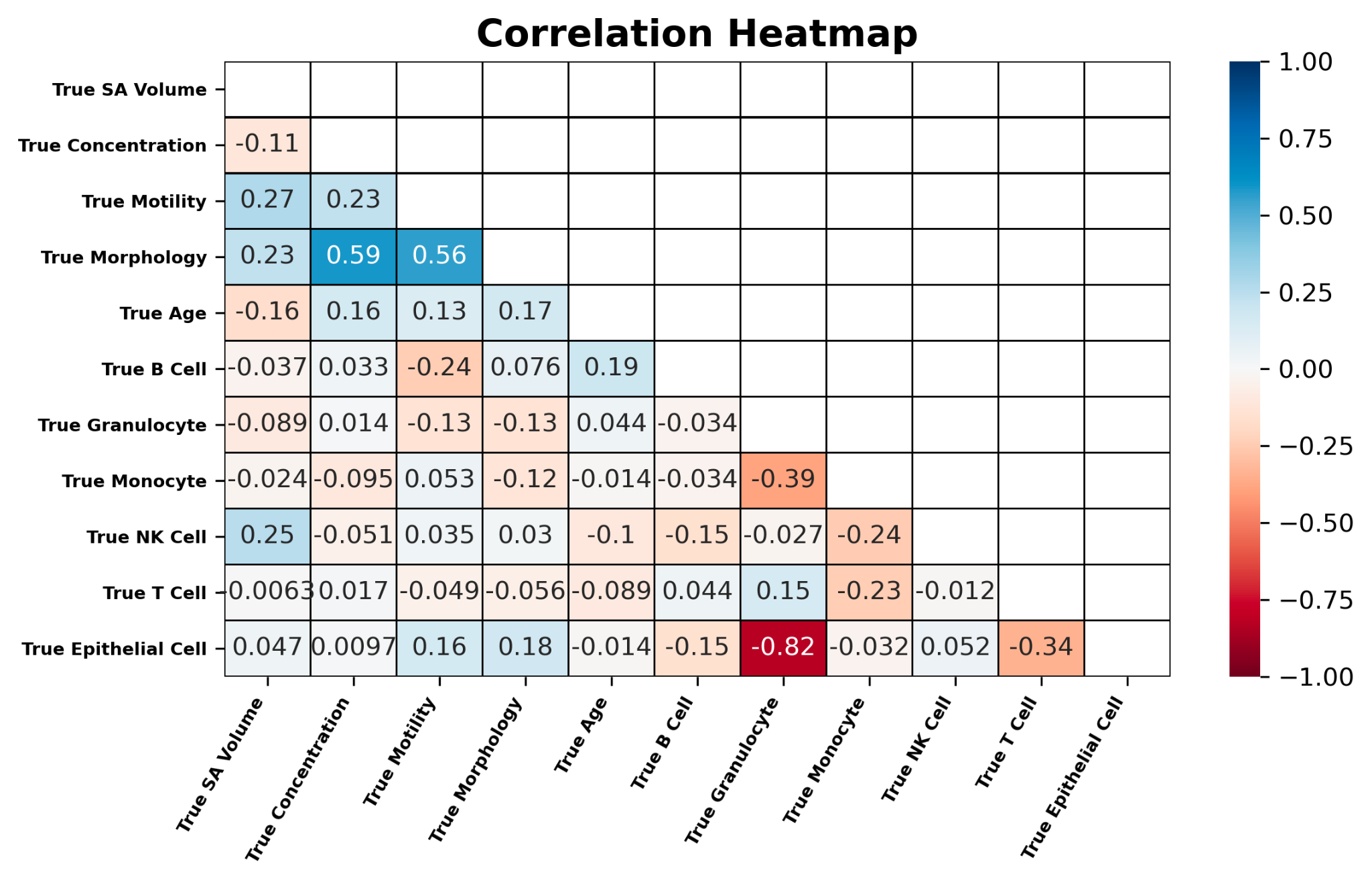


Supplementary Figure 3

**A**

**
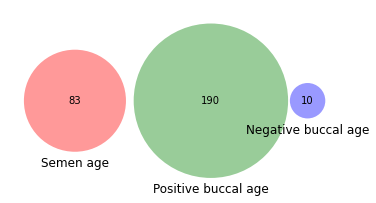
**

Supplementary Table 1

| **GO terms** | **P-value** | **FDR** | **Genes** |
| --- | --- | --- | --- |
| [cytokine activity](http://amigo.geneontology.org/amigo/term/GO:0005125) | 7.563E-05 | 7.813E-02 | [219](http://go.cistrome.org/gogenes?uid=1690257880&t=mf&go=GO:0005125) |
| [cytokine receptor binding](http://amigo.geneontology.org/amigo/term/GO:0005126) | 1.715E-04 | 8.857E-02 | [281](http://go.cistrome.org/gogenes?uid=1690257880&t=mf&go=GO:0005126) |
| [chemokine activity](http://amigo.geneontology.org/amigo/term/GO:0008009) | 4.789E-04 | 1.649E-01 | [48](http://go.cistrome.org/gogenes?uid=1690257880&t=mf&go=GO:0008009) |
| [chemokine receptor binding](http://amigo.geneontology.org/amigo/term/GO:0042379) | 8.549E-04 | 1.838E-01 | [63](http://go.cistrome.org/gogenes?uid=1690257880&t=mf&go=GO:0042379) |
| [solute:proton symporter activity](http://amigo.geneontology.org/amigo/term/GO:0015295) | 8.897E-04 | 1.838E-01 | [26](http://go.cistrome.org/gogenes?uid=1690257880&t=mf&go=GO:0015295) |

Supplementary Table 2

| **GO terms** | **P-value** | **FDR** | **Genes** |
| --- | --- | --- | --- |
| [immune response](http://amigo.geneontology.org/amigo/term/GO:0006955) | 1.944E-08 | 5.443E-05 | [927](http://go.cistrome.org/gogenes?uid=1690257880&t=bp&go=GO:0006955) |
| [defense response](http://amigo.geneontology.org/amigo/term/GO:0006952) | 2.377E-08 | 5.443E-05 | [1064](http://go.cistrome.org/gogenes?uid=1690257880&t=bp&go=GO:0006952) |
| [cytokine-mediated signaling pathway](http://amigo.geneontology.org/amigo/term/GO:0019221) | 3.395E-08 | 5.443E-05 | [610](http://go.cistrome.org/gogenes?uid=1690257880&t=bp&go=GO:0019221) |
| [regulation of viral genome replication](http://amigo.geneontology.org/amigo/term/GO:0045069) | 2.251E-07 | 2.707E-04 | [89](http://go.cistrome.org/gogenes?uid=1690257880&t=bp&go=GO:0045069) |
| [negative regulation of viral genome replication](http://amigo.geneontology.org/amigo/term/GO:0045071) | 1.412E-06 | 1.180E-03 | [53](http://go.cistrome.org/gogenes?uid=1690257880&t=bp&go=GO:0045071) |
| [response to interferon-gamma](http://amigo.geneontology.org/amigo/term/GO:0034341) | 1.579E-06 | 1.180E-03 | [114](http://go.cistrome.org/gogenes?uid=1690257880&t=bp&go=GO:0034341) |
| [defense response to virus](http://amigo.geneontology.org/amigo/term/GO:0051607) | 1.717E-06 | 1.180E-03 | [190](http://go.cistrome.org/gogenes?uid=1690257880&t=bp&go=GO:0051607) |
| [regulation of viral life cycle](http://amigo.geneontology.org/amigo/term/GO:1903900) | 2.565E-06 | 1.542E-03 | [138](http://go.cistrome.org/gogenes?uid=1690257880&t=bp&go=GO:1903900) |
| [response to cytokine](http://amigo.geneontology.org/amigo/term/GO:0034097) | 3.062E-06 | 1.570E-03 | [546](http://go.cistrome.org/gogenes?uid=1690257880&t=bp&go=GO:0034097) |
| [negative regulation of viral entry into host cell](http://amigo.geneontology.org/amigo/term/GO:0046597) | 3.533E-06 | 1.570E-03 | [19](http://go.cistrome.org/gogenes?uid=1690257880&t=bp&go=GO:0046597) |

Supplementary Table 3

| **GO terms** | **P-value** | **FDR** | **Genes** |
| --- | --- | --- | --- |
| [chylomicron](http://amigo.geneontology.org/amigo/term/GO:0042627) | 2.770E-04 | 1.831E-01 | [14](http://go.cistrome.org/gogenes?uid=1690257956&t=cc&go=GO:0042627) |
| [very-low-density lipoprotein particle](http://amigo.geneontology.org/amigo/term/GO:0034361) | 5.756E-04 | 1.902E-01 | [20](http://go.cistrome.org/gogenes?uid=1690257956&t=cc&go=GO:0034361) |

Supplementary Table 4

| **GO terms** | **P-value** | **FDR** | **Genes** |
| --- | --- | --- | --- |
| [adenosine deaminase activity](http://amigo.geneontology.org/amigo/term/GO:0004000) | 7.894E-05 | 7.793E-02 | [12](http://go.cistrome.org/gogenes?uid=1690257956&t=mf&go=GO:0004000) |
| [receptor serine/threonine kinase binding](http://amigo.geneontology.org/amigo/term/GO:0033612) | 1.509E-04 | 7.793E-02 | [23](http://go.cistrome.org/gogenes?uid=1690257956&t=mf&go=GO:0033612) |
| [deaminase activity](http://amigo.geneontology.org/amigo/term/GO:0019239) | 6.055E-04 | 1.772E-01 | [32](http://go.cistrome.org/gogenes?uid=1690257956&t=mf&go=GO:0019239) |
| [hydrolase activity, acting on carbon-nitrogen (but not peptide) bonds, in cyclic amidines](http://amigo.geneontology.org/amigo/term/GO:0016814) | 6.862E-04 | 1.772E-01 | [34](http://go.cistrome.org/gogenes?uid=1690257956&t=mf&go=GO:0016814) |

Supplementary Table 5

| **GO terms** | **P-value** | **FDR** | **Genes** |
| --- | --- | --- | --- |
| [positive regulation of type I interferon-mediated signaling pathway](http://amigo.geneontology.org/amigo/term/GO:0060340) | 8.187E-06 | 3.829E-02 | [13](http://go.cistrome.org/gogenes?uid=1690257956&t=bp&go=GO:0060340) |
| [regulation of type I interferon production](http://amigo.geneontology.org/amigo/term/GO:0032479) | 1.592E-05 | 3.829E-02 | [113](http://go.cistrome.org/gogenes?uid=1690257956&t=bp&go=GO:0032479) |
| [MyD88-independent toll-like receptor signaling pathway](http://amigo.geneontology.org/amigo/term/GO:0002756) | 4.343E-05 | 5.509E-02 | [26](http://go.cistrome.org/gogenes?uid=1690257956&t=bp&go=GO:0002756) |
| [toll-like receptor 3 signaling pathway](http://amigo.geneontology.org/amigo/term/GO:0034138) | 5.253E-05 | 5.509E-02 | [11](http://go.cistrome.org/gogenes?uid=1690257956&t=bp&go=GO:0034138) |
| [regulation of type I interferon-mediated signaling pathway](http://amigo.geneontology.org/amigo/term/GO:0060338) | 5.727E-05 | 5.509E-02 | [30](http://go.cistrome.org/gogenes?uid=1690257956&t=bp&go=GO:0060338) |
| [negative regulation of leukocyte apoptotic process](http://amigo.geneontology.org/amigo/term/GO:2000107) | 1.547E-04 | 9.934E-02 | [49](http://go.cistrome.org/gogenes?uid=1690257956&t=bp&go=GO:2000107) |
| [regulation of cholesterol esterification](http://amigo.geneontology.org/amigo/term/GO:0010872) | 1.755E-04 | 9.934E-02 | [11](http://go.cistrome.org/gogenes?uid=1690257956&t=bp&go=GO:0010872) |
| [toll-like receptor signaling pathway](http://amigo.geneontology.org/amigo/term/GO:0002224) | 1.815E-04 | 9.934E-02 | [86](http://go.cistrome.org/gogenes?uid=1690257956&t=bp&go=GO:0002224) |
| [positive regulation of response to cytokine stimulus](http://amigo.geneontology.org/amigo/term/GO:0060760) | 1.960E-04 | 9.934E-02 | [55](http://go.cistrome.org/gogenes?uid=1690257956&t=bp&go=GO:0060760) |
| [phospholipid efflux](http://amigo.geneontology.org/amigo/term/GO:0033700) | 2.104E-04 | 9.934E-02 | [12](http://go.cistrome.org/gogenes?uid=1690257956&t=bp&go=GO:0033700) |

Supplementary Table 6

| **GO terms** | **P-value** | **FDR** | **Genes** |
| --- | --- | --- | --- |
| [inflammasome complex](http://amigo.geneontology.org/amigo/term/GO:0061702) | 1.738E-04 | 1.149E-01 | [14](http://go.cistrome.org/gogenes?uid=1690952835&t=cc&go=GO:0061702) |
| [chylomicron](http://amigo.geneontology.org/amigo/term/GO:0042627) | 5.733E-04 | 1.568E-01 | [14](http://go.cistrome.org/gogenes?uid=1690952835&t=cc&go=GO:0042627) |
| [cytoplasmic region](http://amigo.geneontology.org/amigo/term/GO:0099568) | 8.047E-04 | 1.568E-01 | [249](http://go.cistrome.org/gogenes?uid=1690952835&t=cc&go=GO:0099568) |
| [cell cortex](http://amigo.geneontology.org/amigo/term/GO:0005938) | 9.615E-04 | 1.568E-01 | [167](http://go.cistrome.org/gogenes?uid=1690952835&t=cc&go=GO:0005938) |
| [cell body](http://amigo.geneontology.org/amigo/term/GO:0044297) | 1.224E-03 | 1.568E-01 | [428](http://go.cistrome.org/gogenes?uid=1690952835&t=cc&go=GO:0044297) |
| [plasma membrane bounded cell projection part](http://amigo.geneontology.org/amigo/term/GO:0120038) | 1.609E-03 | 1.568E-01 | [1438](http://go.cistrome.org/gogenes?uid=1690952835&t=cc&go=GO:0120038) |
| [cytoskeletal part](http://amigo.geneontology.org/amigo/term/GO:0044430) | 2.244E-03 | 1.568E-01 | [1641](http://go.cistrome.org/gogenes?uid=1690952835&t=cc&go=GO:0044430) |
| [very-low-density lipoprotein particle](http://amigo.geneontology.org/amigo/term/GO:0034361) | 2.248E-03 | 1.568E-01 | [20](http://go.cistrome.org/gogenes?uid=1690952835&t=cc&go=GO:0034361) |
| [synaptic cleft](http://amigo.geneontology.org/amigo/term/GO:0043083) | 2.375E-03 | 1.568E-01 | [14](http://go.cistrome.org/gogenes?uid=1690952835&t=cc&go=GO:0043083) |
| [endoplasmic reticulum-Golgi intermediate compartment membrane](http://amigo.geneontology.org/amigo/term/GO:0033116) | 2.531E-03 | 1.568E-01 | [70](http://go.cistrome.org/gogenes?uid=1690952835&t=cc&go=GO:0033116) |

Supplementary Table 7

| **GO terms** | **P-value** | **FDR** | **Genes** |
| --- | --- | --- | --- |
| [tau protein binding](http://amigo.geneontology.org/amigo/term/GO:0048156) | 1.023E-04 | 1.057E-01 | [45](http://go.cistrome.org/gogenes?uid=1690952835&t=mf&go=GO:0048156) |

Supplementary Table 8

| **GO terms** | **P-value** | **FDR** | **Genes** |
| --- | --- | --- | --- |
| [negative regulation of viral entry into host cell](http://amigo.geneontology.org/amigo/term/GO:0046597) | 2.995E-07 | 1.441E-03 | [19](http://go.cistrome.org/gogenes?uid=1690952835&t=bp&go=GO:0046597) |
| [regulation of viral entry into host cell](http://amigo.geneontology.org/amigo/term/GO:0046596) | 2.154E-06 | 5.181E-03 | [30](http://go.cistrome.org/gogenes?uid=1690952835&t=bp&go=GO:0046596) |
| [cytokine-mediated signaling pathway](http://amigo.geneontology.org/amigo/term/GO:0019221) | 6.078E-06 | 9.744E-03 | [610](http://go.cistrome.org/gogenes?uid=1690952835&t=bp&go=GO:0019221) |
| [response to interferon-alpha](http://amigo.geneontology.org/amigo/term/GO:0035455) | 3.186E-05 | 3.831E-02 | [20](http://go.cistrome.org/gogenes?uid=1690952835&t=bp&go=GO:0035455) |
| [type I interferon signaling pathway](http://amigo.geneontology.org/amigo/term/GO:0060337) | 6.404E-05 | 6.161E-02 | [66](http://go.cistrome.org/gogenes?uid=1690952835&t=bp&go=GO:0060337) |
| [regulation of cytoskeleton organization](http://amigo.geneontology.org/amigo/term/GO:0051493) | 1.001E-04 | 6.823E-02 | [516](http://go.cistrome.org/gogenes?uid=1690952835&t=bp&go=GO:0051493) |
| [negative regulation of viral life cycle](http://amigo.geneontology.org/amigo/term/GO:1903901) | 1.126E-04 | 6.823E-02 | [77](http://go.cistrome.org/gogenes?uid=1690952835&t=bp&go=GO:1903901) |
| [response to interferon-beta](http://amigo.geneontology.org/amigo/term/GO:0035456) | 1.266E-04 | 6.823E-02 | [30](http://go.cistrome.org/gogenes?uid=1690952835&t=bp&go=GO:0035456) |
| [negative regulation of cell population proliferation](http://amigo.geneontology.org/amigo/term/GO:0008285) | 1.444E-04 | 6.823E-02 | [668](http://go.cistrome.org/gogenes?uid=1690952835&t=bp&go=GO:0008285) |
| [nuclear envelope organization](http://amigo.geneontology.org/amigo/term/GO:0006998) | 1.595E-04 | 6.823E-02 | [44](http://go.cistrome.org/gogenes?uid=1690952835&t=bp&go=GO:0006998) |
